# Propensity-score matched outcomes of resection of stage IV primary colon cancer with and without simultaneous resection of liver metastases

**DOI:** 10.1007/s13304-024-01832-4

**Published:** 2024-04-03

**Authors:** Sameh Hany Emile, Nir Horesh, Zoe Garoufalia, Rachel Gefen, Peige Zhou, Steven D. Wexner

**Affiliations:** 1 Ellen Leifer Shulman and Steven Shulman Digestive Disease Center, Cleveland Clinic Florida, 2950 Cleveland Clinic Blvd., Weston, FL 33179 USA; 2 Colorectal Surgery Unit, General Surgery Department, Mansoura University Hospitals, Mansoura, Egypt; 3 Department of Surgery and Transplantation, Sheba Medical Center, Ramat-Gan, Israel; 4 Department of General Surgery, Hadassah Medical Organization and Faculty of Medicine, Hebrew University of Jerusalem, Jerusalem, Israel; 5 Georgia Colon and Rectal Surgical Associates, Northside Hospital, Atlanta, Georgia

**Keywords:** Colon cancer, Resection, Liver metastases, Simultaneous, Propensity-score

## Abstract

There is controversy in the best management of colorectal cancer liver metastasis (CLM). This study aimed to compare short-term and survival outcomes of simultaneous resection of CLM and primary colon cancer compared to resection of only colon cancer. This retrospective matched cohort study included patients from the National Cancer Database (2015–2019) with stage IV colon adenocarcinoma and synchronous liver metastases who underwent colectomy. Patients were divided into two groups: colectomy-only (resection of primary colon cancer only) and colectomy-plus (simultaneous resection of primary colon cancer and liver metastases). The groups were matched using the propensity score method. The primary outcome was short-term mortality and readmission. Secondary outcomes were conversion, hospital stay, surgical margins, and overall survival. 4082 (37.6%) of 10,862 patients underwent simultaneous resection of primary colon cancer and liver metastases. After matching, 2038 patients were included in each group. There were no significant differences between the groups in 30-days mortality (3.1% vs 3.8%, *p* = 0.301), 90-days (6.6% vs 7.7%, *p* = 0.205) mortality, 30-days unplanned readmission (7.2% vs 5.3%, *p* = 0.020), or conversion to open surgery (15.5% vs. 13.8%, *p* = 0.298). Patients in the colectomy plus group had a higher rate of lower incidence of positive surgical margins (13.2% vs. 17.2%, *p* = 0.001) and longer overall survival (median: 41.5 vs 28.4 months, *p* < 0.001). Synchronous resection of CLM did not increase the rates of short-term mortality, readmission, conversion from minimally invasive to open surgery, or hospital stay and was associated with a lower incidence of positive surgical margins.

**Supplementary Information:**

The online version contains supplementary material available at 10.1007/s13304-024-01832-4.

## Background

Liver metastasis is the most common form of distant spread of colon cancer. It has been estimated that one-quarter of patients with colorectal cancer (CRC) will develop liver metastases during the course of their disease [1, 2]. The presence of colorectal liver metastases (CLM) is typically associated with poorer prognosis. Although the 5-years overall survival (OS) of patients with hepatic CLM doubled from 30 to 60% during the past decades [3], the oncologic outcomes remain sub-optimal.

According to a population-based study, CLM are more often diagnosed in left-sided colon cancers, however, they tend to be more extensive in right-sided cancers which may explain the worse OS and prognosis [2]. CLM can be synchronous or metachronous depending on whether they were detected at or after the time of diagnosis of the primary cancer. Metachronous CLM are usually discovered within 3–6 months after the diagnosis of primary CRC [4, 5]. CLM are assessed with imaging techniques including ultrasound, computed tomography (CT) scanning, magnetic resonance imaging (MRI), and positron emission tomography (PET-CT). This assessment is crucial to obtain information on the location, size, and vascular anatomy of the liver metastases which directly guides the treatment strategy [6–8].

Treatment of CLM entails a variety of options, including radiofrequency ablation, chemotherapy, portal vein embolization, and surgical resection. Each treatment modality has its set of indications. Treatment of synchronous liver metastases, concurrent with, before, or after resection of the primary colon cancer, may improve OS. A meta-analysis showed that simultaneous resection of CLM confers equivalent long-term prognosis to that of staged resection and is overall safe with similar odds of total and organ-specific complications and perioperative mortality [9].

The present study aimed to compare the short-term and survival outcomes of patients with synchronous CLM who underwent or did not undergo simultaneous resection of CLM with the primary colon cancer. The study hypothesis was that simultaneous resection of CLM, albeit being a complex procedure, does not increase the short-term mortality or compromise the short-term outcomes of colectomy when compared to resection of the primary colon cancer only.

## Patients and methods

### Study design and data source

This study was a retrospective cohort analysis of patients with stage IV colon adenocarcinoma with synchronous liver metastases who underwent colectomy. Data used in the study were derived from the National Cancer Database (NCDB) between 2015 and 2019. The NCDB is a joint project of the Commission on Cancer (CoC) of the American College of Surgeons and the American Cancer Society. This clinical oncology national database includes hospital registry data from > 1500 Commission on Cancer (CoC) accredited hospitals in the United States. It should be noted that the NCDB and the hospitals participating in the CoC NCDB herein have not verified and are not responsible for the statistical validity of the data analysis or the conclusions derived by the authors. Ethics committee approval and written consent to participate in the study were not required given that the study was retrospective and was based on a public database that includes de-identified patient data.

### Study population

The NCDB Participant User File (PUF) was reviewed and interpreted using the relevant PUF dictionary. We included patients who were diagnosed with stage IV colon adenocarcinomas (ICDO-3 code 8140/3, 8480–8481/3, 8490/3) who had synchronous hepatic metastases and no other organ metastases. The exclusion criteria were patients with appendiceal cancers, patients with stage I–III colon cancers or with unknown clinical stage, patients with other organ metastases, and patients who did not have colectomy or if their surgery type was not specified. Colectomy included segmental resection, hemicolectomy, subtotal colectomy, total colectomy, proctocolectomy, and non-specified colectomy. The study was reported consistent with reporting guidelines for propensity-score matched analyses [10].

### Data points

The following data were collected and used for the analysis:

- Baseline characteristics: age, sex, race, Charlson score, clinical TNM stage, insurance status, and tumor location.
- Pathologic parameters: tumor histology, grade, lymphovascular invasion, MSI status, and KRAS status.
- Treatment details: chemotherapy, immunotherapy, sequencing of systemic therapy, type and approach of colectomy, and days from diagnosis to surgery.
- Outcomes: conversion to open surgery, surgical margins, number of examined lymph nodes, 30- and 90-day mortality, 30-day readmission, and overall survival (OS).

### Study outcomes

The primary outcomes were 30- and 90-day mortality and 30-day readmission. Secondary endpoints included hospital stay, conversion to open surgery, surgical margins, and OS.

### Data analysis

The cohort was divided into two groups: colectomy-only (only resection of the primary colon cancer) and colectomy-plus (simultaneous resection of both the primary colon cancer and liver metastases). The selection of variables for PSM was mainly based on clinical judgment of which covariates could impact the primary outcome, specifically if they showed an imbalance in the original cohort, implied by a standardized mean difference (SMD) > 1. The nearest neighbor 1:1 propensity-score matching with a caliper of 0.2 was used as it was suggested to be the optimal caliper width for propensity-score matching when estimating differences in means and differences in proportions in observational studies [11]. A secondary propensity-score matched analysis with a caliper of 0.1 matching for all possible covariates was conducted.

The true population of interest included patients with colonic adenocarcinomas with synchronous hepatic metastases and no other organ metastases who underwent resection of primary colonic cancer with or without resection of liver metastases. The population was selected regardless of the number and location of liver metastases or the extent of liver resection that was unknown. The PSM analysis involved the average effect of the treatment on the treated (ATT) since it aimed to assess the effect of simultaneous colonic and hepatic resection on short-term outcomes for patients who received or did not receive the intervention.

Statistical analyses were performed using EZR™ (version 1.55), R software (version 4.1.2), and SPSS™ version 23 (IBM Corp). Continuous data were expressed as mean and standard deviation when normally distributed or otherwise as median and interquartile range (IQR). Student-t test or Mann–Whitney test was used to process continuous variables. Categorical data were expressed in the form of numbers and proportions and were analyzed using the Fisher exact test or Chi-Square test. Kaplan–Meier statistics and log-rank tests were used to detect differences in OS between the two groups. Bonferroni adjustment was used to adjust the threshold for statistical significance to less than 0.012 as three primary endpoints were reported. Sensitivity analyses using the Rosenbaum and Mantel–Haenszel methods were conducted. Gamma values indicated the setting of sensitivity parameter used and the lower and upper p-values represent the lower and upper bound of the confidence interval for the Mantel–Haenszel statistic.

## Results

### Patient characteristics

**Fig. 1:**
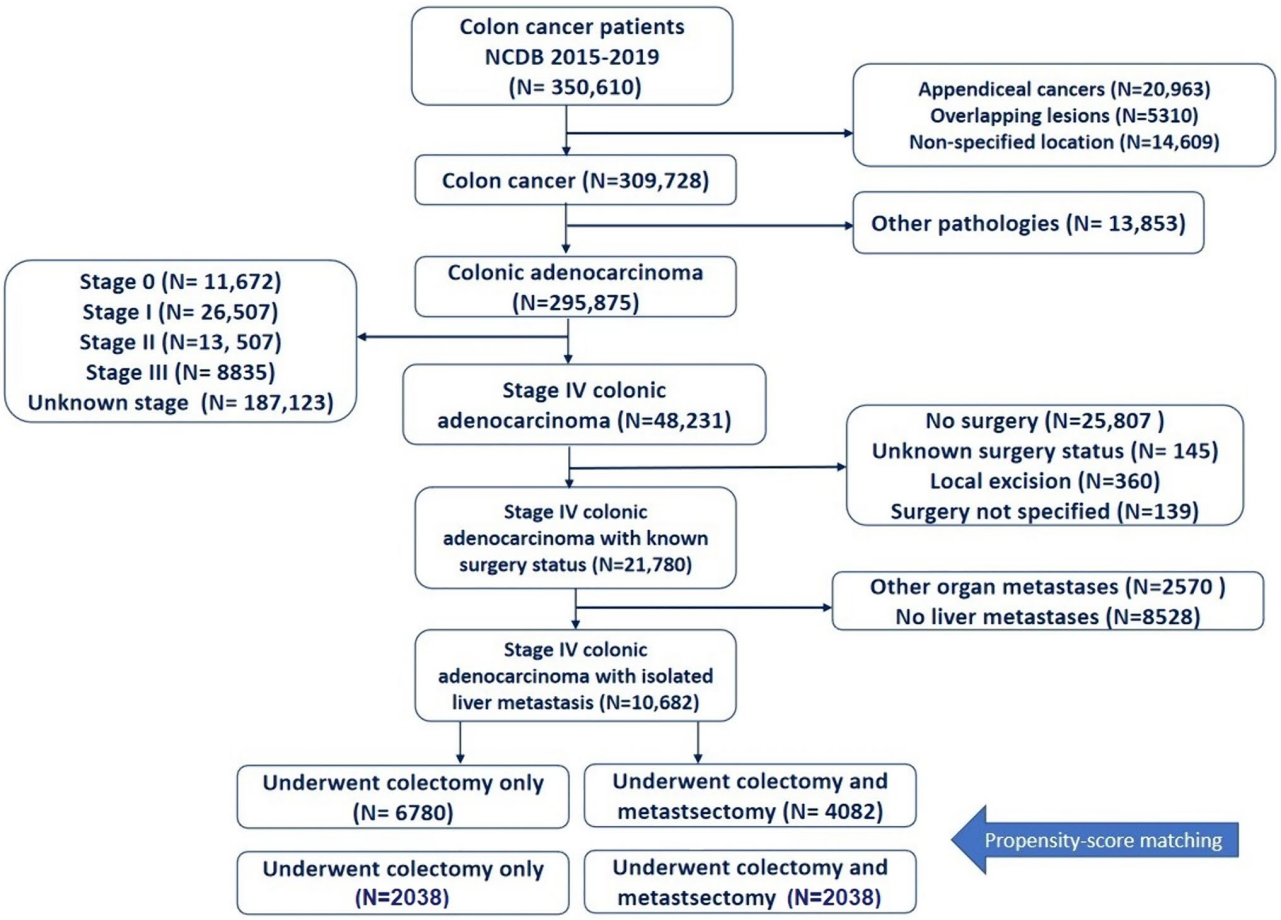
Flow chart for patient inclusion in the study

After screening the records of 48,231 patients with stage IV colon adenocarcinoma, 10,862 patients were included (Fig. 1). The mean age of patients was 61.2 ± 13.6 years. Male patients accounted for 55.1% of the studied cohort. The majority of patients were White (78.4%), had a Charlson score of 0 (74.7%), and were insured by Medicare (42.8%) or private insurance (42.5%). Tumors were equally located in the right and left colon, while 10% were in the transverse colon. More than half (52.5%) of colectomies were conducted via a laparotomy and 47.5% were via a minimally invasive approach. The majority of (57.2%) resections were hemicolectomies or subtotal colectomies, whereas 38.1% were segmental resections. A summary of the cohort characteristics is shown in Table 1.

**Table 1:** Characteristics of the study cohort

Factor	Group	Overall
Number		10,862
Mean age in years (SD)		62.16 (13.64)
Sex (%)	Male	5981 (55.1)
	Female	4881 (44.9)
Race (%)	White	8465 (78.4)
	Black	1752 (16.2)
	Asian	358 (3.3)
	American Indian	48 (0.4)
	Other	169 (1.6)
Charlson Deyo score (%)	0	8114 (74.7)
	1	1762 (16.2)
	2	555 (5.1)
	3	431 (4.0)
Insurance (%)	Medicaid	1066 (9.9)
	Medicare	4594 (42.8)
	Other government	136 (1.3)
	Private insurance	4561 (42.5)
	Not insured	371 (3.5)
Tumor location (%)	Right	4862 (44.8)
	Left	4918 (45.3)
	Transverse colon	1082 (10.0)
Systemic treatment (%)	No systemic therapy	2182 (20.2)
	Neoadjuvant	1448 (13.4)
	Adjuvant	5814 (53.9)
	Neoadjuvant and adjuvant	1316 (12.2)
	Intraoperative	32 (0.3)
Surgical approach (%)	Open	4614 (52.5)
	Laparoscopic	3385 (38.5)
	Robotic-assisted	797 (9.1)
Type of colectomy (%)	Segmental resection	4144 (38.1)
	Subtotal colectomy/hemicolectomy	6211 (57.2)
	Total colectomy	289 (2.7)
	Total proctocolectomy	43 (0.4)
	Non specified colectomy	175 (1.6)
Metastsectomy (%)	Without metastectomy	6780 (62.4)
	With metastectomy	4082 (37.6)

### Matching

Resection of only the primary colon cancer was done in 6780 (62.4%) patients whereas simultaneous resection of colon cancer and liver metastases was undertaken in 4082 (37.6%) patients. Patients in the colectomy-only group were older (63.7 ± 13.6 vs 59.6 ± 13.3 years), more often male (56% vs 53.5%), more often had a Charlson score of 3 (4.4% vs 3.3%), more often presented with right-sided cancers (45.7% vs 43.2%), had a shorter time before surgery (median: 10 vs 30 days), more often underwent minimally invasive resection (49.7% vs 43.5%), and less often were treated with chemotherapy (48.6% vs 50.7%) and immunotherapy (34.1% vs 40.6%). The matching criteria included age, sex, Charlson score, tumor location, time before surgery, surgical approach, and systemic therapy. After matching, 2038 patients were included in each group (Table 2).

**Table 2:** Baseline comparison between colectomy only and colectomy with metastasectomy

Factor	Group	Colectom-only (*n* = 6780)	Colectomy-plus (*n* = 4082)	SMD	Colectomy-only (*n* = 2038)	Colectomy-plus (*n* = 2038)	SMD
Mean age in years (SD)		63.73 (13.59)	59.56 (13.32)	0.31	60.70 (13.36)	61.08 (13.53)	0.028
Sex (%)	Male	3796 (56.0)	2185 (53.5)	0.049	1083 (53.1)	1087 (53.3)	0.004
	Female	2984 (44.0)	1897 (46.5)		955 (46.9)	951 (46.7)	
Race (%)	White	5254 (78.0)	3211 (79.1)	0.045	1603 (78.7)	1608 (78.9)	0.012
	Black	1130 (16.8)	622 (15.3)		338 (16.6)	338 (16.6)	
	Asian	213 (3.2)	145 (3.6)		65 (3.2)	61 (3.0)	
	American Indian	30 (0.4)	18 (0.4)		8 (0.4)	8 (0.4)	
	Other	108 (1.6)	61 (1.5)		24 (1.2)	23 (1.1)	
Charlson score (%)	0	4990 (73.6)	3124 (76.5)	0.079	1522 (74.7)	1519 (74.5)	0.04
	1	1149 (16.9)	613 (15.0)		350 (17.2)	335 (16.4)	
	2	346 (5.1)	209 (5.1)		95 (4.7)	111 (5.4)	
	3	295 (4.4)	136 (3.3)		71 (3.5)	73 (3.6)	
Insurance (%)	Medicaid	666 (10.0)	400 (9.9)	0.25	217 (10.8)	194 (9.6)	0.124
	Medicare	3113 (46.5)	1481 (36.7)		806 (40.1)	835 (41.4)	
	Other government	76 (1.1)	60 (1.5)		21 (1.0)	24 (1.2)	
	Private insurance	2561 (38.3)	2000 (49.5)		870 (43.3)	911 (45.2)	
	Not insured	273 (4.1)	98 (2.4)		96 (4.8)	53 (2.6)	
Tumor location (%)	Right colon	3099 (45.7)	1763 (43.2)	0.069	939 (46.1)	931 (45.7)	0.008
	Left colon	2983 (44.0)	1935 (47.4)		903 (44.3)	909 (44.6)	
	Transverse colon	698 (10.3)	384 (9.4)		196 (9.6)	198 (9.7)	
Histology (%)	Adenocarcinoma	6371 (94.0)	3819 (93.6)	0.035	1906 (93.5)	1895 (93.0)	0.058
	Mucinous adenocarcinoma	367 (5.4)	245 (6.0)		117 (5.7)	135 (6.6)	
	Signet-ring cell carcinoma	42 (0.6)	18 (0.4)		15 (0.7)	8 (0.4)	
Grade (%)	Well-differentiated	361 (6.1)	170 (5.6)	0.1	141 (6.9)	96 (4.7)	0.124
	Moderately differentiated	4039 (68.6)	2227 (73.0)		1404 (68.9)	1503 (73.7)	
	Poorly differentiated	1299 (22.1)	564 (18.5)		431 (21.1)	376 (18.4)	
	Undifferentiated	190 (3.2)	88 (2.9)		62 (3.0)	63 (3.1)	
Lymphovascular invasion (%)	No	2206 (36.9)	1380 (40.3)	0.071	716 (38.4)	726 (39.1)	0.014
	Yes	3777 (63.1)	2042 (59.7)		1149 (61.6)	1132 (60.9)	
KRAS (%)	Mutated	715 (47.1)	516 (47.6)	0.012	253 (43.5)	314 (47.9)	0.087
	Wild	804 (52.9)	567 (52.4)		328 (56.5)	342 (52.1)	
MSI (%)	Negative	1335 (85.1)	842 (85.3)	0.019	501 (85.1)	520 (84.7)	0.087
	Positive	234 (14.9)	145 (14.7)		88 (14.9)	94 (15.3)	
Median time between diagnosis and definitive surgery		10 [2, 39]	30 [4, 152]	0.409	14 [2, 47]	16 [3, 49]	< 0.001
Surgical approach (%)	Open	2852 (50.3)	1762 (56.4)	0.125	1072 (52.6)	1109 (54.4)	0.039
	Laparoscopic	2291 (40.4)	1094 (35.0)		785 (38.5)	763 (37.4)	
	Robotic assisted	531 (9.4)	266 (8.5)		181 (8.9)	166 (8.1)	
Type of colectomy (%)	Segmental resection	2590 (38.2)	1554 (38)	0.101	783 (38.4)	732 (35.9)	0.1
	Subtotal colectomy/hemicolectomy	3890 (57.4)	2321 (56.9)		1152 (56.6)	1207 (59.2)	
	Total colectomy	182 (2.6)	107 (2.7)		59 (2.9)	57 (2.8)	
	Total proctocolectomy	21 (0.3)	22 (0.6)		9 (0.4)	11 (0.5)	
	Non specified colectomy	97 (1.4)	78 (1.9)		46 (2.2)	39 (1.9)	
Chemotherapy (%)	No	3171 (51.4)	1903 (49.3)	0.042	1091 (53.5)	1073 (52.6)	0.018
	Yes	3004 (48.6)	1960 (50.7)		947 (46.5)	965 (47.4)	
Sequencing of systemic treatment (%)	No systemic therapy	1699 (25.2)	483 (11.9)	0.534	269 (13.2)	252 (12.4)	0.047
	Neoadjuvant	686 (10.2)	762 (18.8)		245 (12.0)	237 (11.6)	
	Adjuvant	3818 (56.7)	1996 (49.2)		1341 (65.8)	1376 (67.5)	
	Neoadjuvant and adjuvant	521 (7.7)	795 (19.6)		177 (8.7)	168 (8.2)	
	Intraoperative	8 (0.1)	24 (0.5)		6 (0.2)	5 (0.2)	

*SMD* standardized mean difference, *SD* standard deviation, *MSI* microsatellite instability

### Outcomes after matching

Based on a significance level of 0.012, there were no significant differences between the two groups in the primary endpoints that included 30-days mortality (3.1% vs 3.8%, *p* = 0.301; OR: 0.81, *p* = 0.266), 90-days mortality (6.6% vs 7.7%, *p* = 0.205; OR: 0.84, *p* = 0.204), and unplanned 30-days readmission (7.2% vs. 5.3%, *p* = 0.020; OR: 1.39, *p* = 0.012). Furthermore, there was no significant differences in conversion to open surgery (15.5% vs. 13.8%, *p* = 0.298; OR: 1.15, *p* = 0.286) and hospital stay (median 6 vs. 5 days, *p* = 0.032), The colectomy-plus group was associated with a significantly lower incidence of positive surgical margins (13.2% vs. 17.2%, *p* = 0.001; OR: 0.73, *p* < 0.001), and greater number of examined lymph nodes (median: 19 vs 18, *p* < 0.001) (Table 3).

**Table 3:** Outcome comparison between colectomy only and colectomy with metastsectomy

Factor	Group	Before PSM			After PSM		
		Colectomy-only (*n* = 6780)	Colectomy-plus (*n* = 4082)	*p*-value	Colectomy-only (*n* = 2038)	Colectomy-plus (*n* = 2038)	*p*-value
Conversion to open (%)	No	2439 (86.4)	1161 (85.4)	0.365	833 (86.2)	785 (84.5)	0.298
	Yes	383 (13.6)	199 (14.6)		133 (13.8)	144 (15.5)	
Median hospital stay in days [IQR]		5 [4, 8]	5 [4, 8]	0.73	5 [4, 8]	6 [4, 8]	**0.032**
30-days readmission (%)	No	6163 (92.3)	3673 (91.8)	0.584	1869 (92.0)	1816 (89.5)	**0.020**
	Planned	130 (1.9)	89 (2.2)		53 (2.6)	65 (3.2)	
	Unplanned	387 (5.8)	237 (6)		109 (5.3)	148 (7.2)	
30-days mortality (%)	No	4954 (94.3)	3056 (97.1)	**< 0.001**	1614 (96.2)	1649 (96.9)	0.301
	Yes	299 (5.7)	90 (2.9)		64 (3.8)	53 (3.1)	
90-days mortality (%)	No	4591 (88.0)	2941 (94.0)	**< 0.001**	1538 (92.3)	1583 (93.4)	0.205
	Yes	627 (12.0)	188 (6.0)		129 (7.7)	112 (6.6)	
Surgical margins (%)	Negative	5546 (83.4)	3534 (88.5)	**< 0.001**	1672 (82.6)	1759 (86.7)	**0.001**
	Positive	1080 (16.3)	448 (11.2)		349 (17.2)	267 (13.2)	
	Not evaluable	20 (0.3)	13 (0.3)		4 (0.2)	4 (0.2)	
Median number of examined regional nodes [IQR]		18 [13, 24]	19 [15, 26]	**< 0.001**	18 [14, 24]	19 [15, 26]	**< 0.001**
Overall survival (%)	Alive	2030 (38.3)	1827 (57.9)	**< 0.001**	683 (40.5)	905 (53.0)	**< 0.001**
	Dead	3277 (61.7)	1330 (42.1)		1003 (59.5)	801 (47.0)	
Median follow up in months [IQR]		20.6 [9.2, 32.3]	29.1 [17.4, 39.8]	**< 0.001**	24.3 [11.8, 35.7]	29.5 [16.2, 40.8]	**< 0.001**

Bold text in the *p* value columns indicates statistical significance

*PSM* propensity score matched, *IQR* interquartile range

Sensitivity analysis according to the Rosenbaum method showed that *p* values for the primary endpoints remained insignificant at different sensitivity levels, indicating robust outcomes that were not sensitive to change caused by unobserved confounders. However, a Mantel–Haenszel sensitivity analysis showed that at a lower gamma of 0.5, the *p* value for the difference in 30-days mortality became significant (*p* = 0.004) (Supplementary Table 1).

**Fig. 2:**
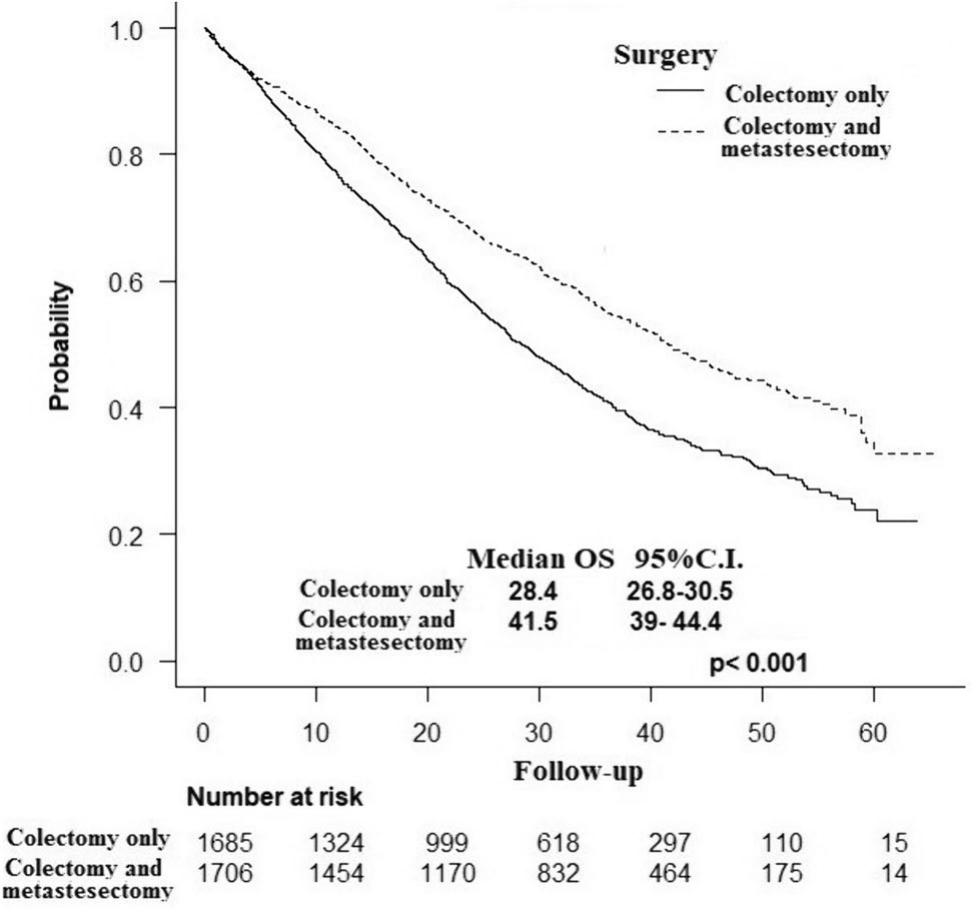
Kaplan Meier curve illustrating the difference in overall survival between the colectomy-only and colectomy-plus groups

**Fig. 3:**
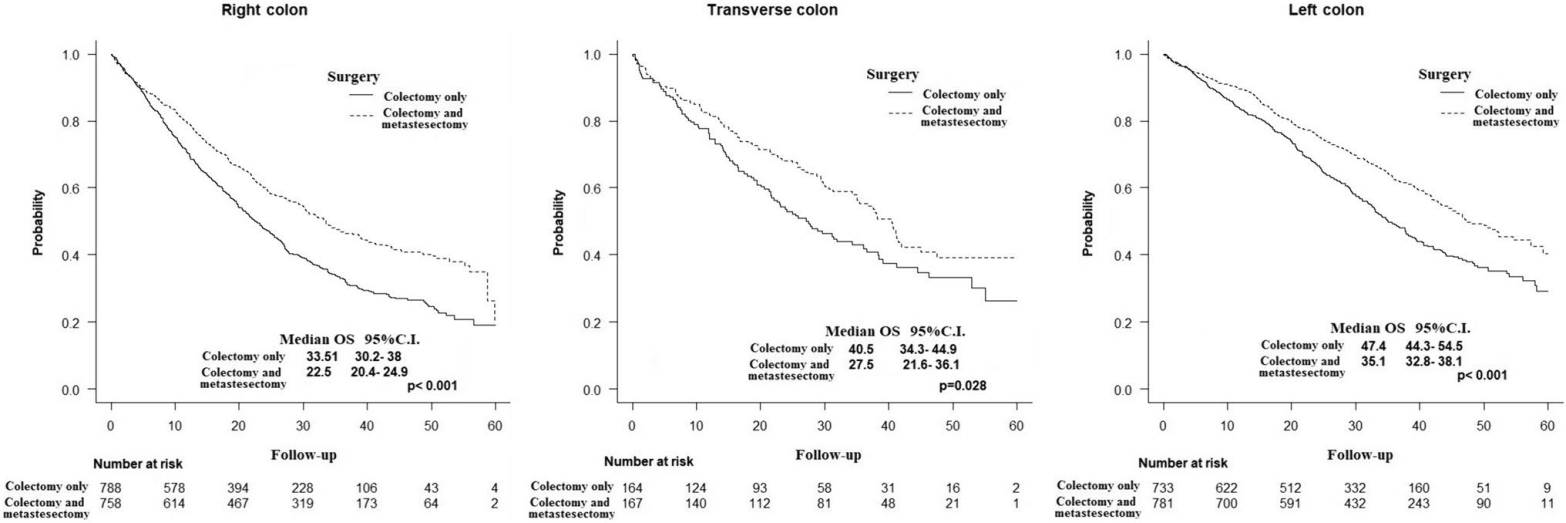
Kaplan Meier curve illustrating the difference in overall survival between the colectomy-only and colectomy-plus groups stratified by tumor location

Patients in the colectomy-plus group had a significantly longer OS (median: 41.5 vs 28.4 months, *p* < 0.001) (Fig. 2). The longer OS in favor of the colectomy-plus group was noted when the survival analysis was stratified by tumor location (right colon: 33.5 vs. 22.5 months, *p* < 0.001; left colon: 40.5 vs, 27.5 months, *p* = 0.028; transverse colon: 47.4 vs. 35.1 months, *p* < 0.001) (Fig. 3).

### Secondary propensity-score matched analysis

A secondary propensity-score matched analysis was conducted, matching for age, sex, race, Charlson score, insurance type, tumor location, tumor histology, tumor, grade, time before surgery, type of colectomy, surgical approach, chemotherapy, and systemic therapy. After matching, 2022 patients were included in each group; both groups had balanced baseline and treatment characteristics with SMD < 1 (Supplementary Table 2).

In the matched cohort, there were no significant differences between the two groups relative to 30-day mortality (3.1% vs 3.1%, *p* = 0.921), 90-day mortality (6.5% vs 7.2%, *p* = 0.491), unplanned 30-day readmission (7.3% vs. 6.8%, *p* = 0.332), conversion to open surgery (15.6% vs. 14.1%, *p* = 0.393), and hospital stay (median 6 vs. 5 days, *p* = 0.039). The colectomy-plus group was associated with a significantly lower incidence of positive surgical margins (13.2% vs. 17.3%, *p* = 0.001), a greater number of examined lymph nodes (median: 19 vs 18, *p* < 0.001), and a significantly longer OS (median: 41.3 vs 28.6 months, *p* < 0.001).

## Discussion

The present study found that resection of CLM at the time of colectomy was not associated with a significant increase in the rate of short-term mortality or conversion to open surgery. Simultaneous resection of CLM was also associated with a lower incidence of positive resection margins. However, it may extend hospital stay by 1 day and increase the rate of unplanned readmission.

Resection of CLM may be done either at the same setting of resection of the primary tumor or at a later stage. There has been debate on the safety of simultaneous versus staged resection of CLM. The American Society of Colon and Rectal Surgeons (ASCRS) guidelines [12] state that, “a single “combined” operation is generally recommended for relatively low complexity operations and sequential or “staged” operations are generally recommended for higher complexity cases”. Although the current literature attests to the safety of simultaneous resection of CLM, the outcome essentially depends on the extent of hepatic resections. Reddy et al. [13] reported that simultaneous colorectal resection and minor liver resection did not significantly increase the overall morbidity and mortality, and, conversely, simultaneous colorectal and major hepatic resections were associated with an increased risk of severe morbidity. Similarly, in a retrospective study [14] on 92 patients with synchronous resectable CLM, 27% of whom underwent major liver resection of ≥ 4 segments, the rate of major complications was 21.7%. The significant predictors of complications were medical comorbidities and major hepatectomy, which increased the complication rate to 40%. A recent meta-analysis [9] concluded that simultaneous resection of the primary tumor and liver metastases can be the first choice in patients with resectable synchronous CLM.

Resection of CLM along with the primary colon cancer in our study was generally safe. The rates of 30- and 90-day mortality, which can be used as a surrogate for major complications, were not significantly increased with simultaneous resection of CLM. The 30-day mortality of the colectomy-plus group was 3.1%, slightly higher than the rate after the second stage of staged resection of CLM that was previously reported to be 2% [15]. Although simultaneous resection may increase short-term morbidity to approximately 30% according to a NSQIP analysis [16], another study [17] found that complications after resection of CLM were associated with low mortality when surgery is undertaken in an enhanced recovery setting. Perhaps the increased incidence of short-term morbidity may explain the longer hospital stay and higher 30-day readmission rate after simultaneous resection of CLM in our study, which is predictable given the higher complexity of the procedure. The lower incidence of positive surgical margins with simultaneous resection of CLM may be related to the loco-regional extent of the disease rather than a direct impact of resection of liver lesions.

It was noteworthy that < 20% of patients who underwent simultaneous resection of CLM in the original cohort of our study received neoadjuvant systemic therapy. This finding indicates that upfront surgery was the preferred strategy for the majority of the CoC-accredited hospitals. There is no clear guidance on the use of neoadjuvant therapy before combined resection of colon cancer and hepatic metastases. According to the ASCRS guidelines, patients with resectable CLM can be either treated with upfront surgery or neoadjuvant chemotherapy followed by surgical resection [12]. The ASCRS recommendation to provide neoadjuvant chemotherapy before surgery was not strong as the evidence was mainly based on the EORTC 40983 trial [18] in which perioperative FOLFOX4, although it improved progression-free survival by 7%, did not significantly improve 5-years OS compared to upfront resection.

In the present study, simultaneous resection of CLM improved OS by more than 1 year on average compared to patients who underwent only colectomy. This finding is expected since simultaneous resection of CLM aims at achieving better oncologic control and a state of no evidence of disease [19]. However, due to the inherent limitations of the database used in the study, we could not know the treatment strategy used for patients with CLM in the colectomy-only group as they may have been treated with systemic therapy alone, radiofrequency ablation, or may have undergone a staged resection at a later date. Given this important limitation, conclusions on long-term survival cannot be firmly drawn. However, the short-term outcomes of the study may add useful information about the safety of simultaneous resection of CLM. Further limitations of the study include its retrospective nature, risk of selection bias, not accounting for confounding factors that were unavailable in the NCDB, and limitations of database studies such as missing data and misclassification. Another limitation that should be noted is the lack of information on the size, number, and location of CLM in both groups. Patients who underwent only colectomy may have had more extensive liver lesions that precluded simultaneous liver resection at the time of colectomy. In addition, we can assume that most patients who underwent simultaneous colonic and liver resection had few accessible hepatic lesions that were amenable to simple resection at the time of colectomy. While this represents an obvious bias in patient selection, it also supports the notion that simultaneous colonic and liver resection could be a safe option in a select group of patients, which is the main objective of our study. The lack of available data on the type of liver resection, operative times, and estimated blood loss is another limitation since limited liver resection is usually associated with less morbidity and blood loss than extensive resections. Moreover, the NCDB  did not include data on preoperative liver imaging, intraoperative hepatic ultrasound, MDT involvement, and hepatobiliary surgeon expertise and availability. The specifics of chemotherapy such as the exact regimen used, dosage, and duration of therapy were also not reported in the NCDB.

## Conclusions

Synchronous resection of CLM did not increase the rates of short-term mortality, readmission, conversion from minimally invasive to open surgery, or hospital stay and was associated with a lower incidence of positive surgical margins. Based upon these data, synchronous resection may be conducted in appropriately selected patients.

### Supplementary Information

Below is the link to the electronic supplementary material.

Supplementary file 1 (DOCX 19 KB)

## Data Availability

Data used in the study will be made available by the corresponding author on reasonable request.
