# Supplementary material for: Propensity-score matched outcomes of resection of stage IV primary colon cancer with and without simultaneous resection of liver metastases
Source: Updates Surg. 2024 Apr 3;76(3):845–53. doi: 10.1007/s13304-024-01832-4 (PMC11130067; doi:10.1007/s13304-024-01832-4)
Supplement: Supplementary file 1 — (DOCX 19 KB) [file 13304_2024_1832_MOESM1_ESM.docx]

**Supplementary table 1.** Results of sensitivity analyses

|  | **Rosenbaum** | | **Mantel Haenszel** | |
| --- | --- | --- | --- | --- |
| **Gamma** | **Lower P-Value** | **Upper P-Value** | **Lower P-Value** | **Upper P-Value** |
| **30-day mortality** | | | | |
| 0.5 | 0.92318183 | 0.9853356 | 0.6966904 | **0.004343564** |
| 1 | 0.05172075 | 0.8087396 | 0.7171375 | 0.362461058 |
| 1.5 | 0.27279784 | 0.2501500 | 0.7447980 | 0.997403087 |
| 2 | 0.63417491 | 0.8829589 | 0.5915612 | 0.894920275 |
| **90-day mortality** | | | | |
| 0.5 | 0.3193538 | 0.53114444 | 0.04640746 | 0.37820292 |
| 1 | 0.4030599 | 0.05466444 | 0.77096992 | 0.41815110 |
| 1.5 | 0.9250974 | 0.98080823 | 0.59187349 | 0.06727268 |
| 2 | 0.8880103 | 0.43597876 | 0.89334002 | 0.53432711 |
| **30-day readmission** | | | | |
| 0.5 | 0.65683510 | 0.4185833 | 0.44263517 | 0.04189153 |
| 1 | 0.59222391 | 0.4720530 | 0.17226918 | 0.55915013 |
| 1.5 | 0.02196199 | 0.1181427 | 0.67982871 | 0.29742709 |
| 2 | 0.69125825 | 0.8293081 | 0.01196706 | 0.35170641 |

**Table 2.** Baseline comparison between colectomy only and colectomy with metastasectomy

| **Factor** | **Group** | **Colectomy only (n=6780)** | **Colectomy plus (n=4082)** | **SMD** | **Colectomy only (n=2022)** | **Colectomy plus (n=2022)** | **SMD** |
| --- | --- | --- | --- | --- | --- | --- | --- |
| **Mean age in years (SD)** | | 63.73 (13.59) | 59.56 (13.32) | 0.31 | 61.18 (13.46) | 61.21 (13.50) | 0.002 |
| **Sex (%)** | **Male** | 3796 (56.0) | 2185 (53.5) | 0.049 | 1089 (53.9) | 1080 (53.4) | 0.009 |
|  | **Female** | 2984 (44.0) | 1897 (46.5) |  | 933 (46.1) | 942 (46.6) |  |
| **Race (%)** | **White** | 5254 (78.0) | 3211 (79.1) | 0.045 | 18 (0.9) | 22 (1.1) | 0.031 |
|  | **Black** | 1130 (16.8) | 622 (15.3) |  | 61 (3.0) | 58 (2.9) |  |
|  | **Asian** | 213 (3.2) | 145 (3.6) |  | 8 (0.4) | 9 (0.4) |  |
|  | **American Indian** | 30 (0.4) | 18 (0.4) |  | 1585 (78.4) | 1599 (79.1) |  |
|  | **Other** | 108 (1.6) | 61 (1.5) |  | 350 (17.3) | 334 (16.5) |  |
| **Charlson score (%)** | **0** | 4990 (73.6) | 3124 (76.5) | 0.079 | 1476 (73.0) | 1496 (74.0) | 0.049 |
|  | **1** | 1149 (16.9) | 613 (15.0) |  | 363 (18.0) | 344 (17.0) |  |
|  | **2** | 346 (5.1) | 209 (5.1) |  | 95 (4.7) | 107 (5.3) |  |
|  | **3** | 295 (4.4) | 136 (3.3) |  | 88 (4.4) | 75 (3.7) |  |
| **Insurance (%)** | **Medicaid** | 666 (10.0) | 400 (9.9) | 0.25 | 185 (9.1) | 195 (9.6) | 0.038 |
|  | **Medicare** | 3113 (46.5) | 1481 (36.7) |  | 853 (42.2) | 841 (41.6) |  |
|  | **Other government** | 76 (1.1) | 60 (1.5) |  | 27 (1.3) | 22 (1.1) |  |
|  | **Private insurance** | 2561 (38.3) | 2000 (49.5) |  | 910 (45.0) | 909 (45.0) |  |
|  | **Not insured** | 273 (4.1) | 98 (2.4) |  | 47 (2.3) | 55 (2.7) |  |
| **Tumor location (%)** | **Right colon** | 3099 (45.7) | 1763 (43.2) | 0.069 | 923 (45.6) | 925 (45.7) | 0.026 |
|  | **Left colon** | 2983 (44.0) | 1935 (47.4) |  | 916 (45.3) | 900 (44.5) |  |
|  | **Transverse colon** | 698 (10.3) | 384 (9.4) |  | 183 (9.1) | 197 (9.7) |  |
| **Histology (%)** | **Adenocarcinoma** | 6371 (94.0) | 3819 (93.6) | 0.035 | 1878 (92.9) | 1881 (93.0) | 0.011 |
|  | **Mucinous adenocarcinoma** | 367 (5.4) | 245 (6.0) |  | 136 (6.7) | 132 (6.5) |  |
|  | **Signet-ring cell carcinoma** | 42 (0.6) | 18 (0.4) |  | 8 (0.4) | 9 (0.4) |  |
| **Grade (%)** | **Well-differentiated** | 361 (6.1) | 170 (5.6) | 0.1 | 129 (6.4) | 99 (4.9) | 0.09 |
|  | **Moderately differentiated** | 4039 (68.6) | 2227 (73.0) |  | 1416 (70.0) | 1491 (73.7) |  |
|  | **Poorly differentiated** | 1299 (22.1) | 564 (18.5) |  | 404 (20.0) | 368 (18.2) |  |
|  | **Undifferentiated** | 190 (3.2) | 88 (2.9) |  | 73 (3.6) | 64 (3.2) |  |
|  | **Positive** | 234 (14.9) | 145 (14.7) |  | 129 (6.4) | 99 (4.9) |  |
| **Median time between diagnosis and definitive surgery** | | 10 [2, 39] | 30 [4, 152] | 0.409 | 13.5 [2, 46.75] | 16 [3, 50] | 0.028 |
| **Surgical approach (%)** | **Open** | 2852 (50.3) | 1762 (56.4) | 0.125 | 1111 (54.9) | 1096 (54.2) | 0.037 |
|  | **Laparoscopic** | 2291 (40.4) | 1094 (35.0) |  | 765 (37.8) | 760 (37.6) |  |
|  | **Robotic-assisted** | 531 (9.4) | 266 (8.5) |  | 146 (7.2) | 166 (8.2) |  |
| **Type of colectomy (%)** | **Segmental resection** | 2590 (38.2) | 1554 (38) | 0.101 | 746 (36.9) | 735 (36.3) | 0.044 |
|  | **Subtotal colectomy/hemicolectomy** | 3890 (57.4) | 2321 (56.9) |  | 1189 (58.4) | 1194 (59.1) |  |
|  | **Total colectomy** | 182 (2.6) | 107 (2.7) |  | 59 (3.0) | 56 (2.8) |  |
|  | **Total proctocolectomy** | 21 (0.3) | 22 (0.6) |  | 11 (0.5) | 9 (0.4) |  |
|  | **Non specified colectomy** | 97 (1.4) | 78 (1.9) |  | 25 (1.2) | 28 (1.4) |  |
| **Chemotherapy (%)** | **No** | 3171 (51.4) | 1903 (49.3) | 0.042 | 1086 (53.7) | 1067 (52.8) | 0.019 |
|  | **Yes** | 3004 (48.6) | 1960 (50.7) |  | 936 (46.3) | 955 (47.2) |  |
| **Sequencing of systemic treatment (%)** | **No systemic therapy** | 1699 (25.2) | 483 (11.9) | 0.534 | 237 (11.7) | 248 (12.3) | 0.036 |
|  | **Neoadjuvant** | 686 (10.2) | 762 (18.8) |  | 227 (11.2) | 236 (11.7) |  |
|  | **Adjuvant** | 3818 (56.7) | 1996 (49.2) |  | 1383 (68.4) | 1356 (67.1) |  |
|  | **Neoadjuvant and adjuvant** | 521 (7.7) | 795 (19.6) |  | 169 (8.4) | 176 (8.7) |  |
|  | **Intraoperative** | 8 (0.1) | 24 (0.5) |  | 6 (0.2) | 6 (0.3) |  |
